# Molecular Identification, Characterization, and Expression Analysis of a Gonadotropin-Releasing Hormone Receptor (GnRH-R) in Pacific Abalone, *Haliotis discus hannai*

**DOI:** 10.3390/molecules25122733

**Published:** 2020-06-12

**Authors:** Md. Rajib Sharker, Zahid Parvez Sukhan, Soo Cheol Kim, Won Kyo Lee, Kang Hee Kho

**Affiliations:** Department of Fisheries Science, College of Fisheries and Ocean Sciences, Chonnam National University, 50 Daehak-ro, Yeosu, Jeonnam 59626, Korea; mrsharker@pstu.ac.bd (M.R.S.); sukhan1026@chonnam.ac.kr (Z.P.S.); greatguy@chonnam.ac.kr (S.C.K.); wklee196@jnu.ac.kr (W.K.L.)

**Keywords:** *Haliotis discus hannai*, GnRH receptor, neural ganglia, gonadal maturation, effective accumulative temperature, in situ hybridization

## Abstract

A full-length cDNA sequence encoding a GnRH receptor was cloned from the pleuropedal ganglion of the Pacific abalone, *Haliotis discus hannai*. The cloned sequence is 1499-bp in length encoding a protein of 460 amino acid residues, with a molecular mass of 52.22 kDa and an isoelectric point (pI) of 9.57. The architecture of HdhGnRH-R gene exhibited key features of G protein-coupled receptors (GPCRs), including seven membrane spanning domains, putative N-linked glycosylation motifs, and phosphorylation sites of serine and threonine residues. It shared 63%, 52%, and 30% sequence identities with *Octopus vulgaris*, *Limulus polyphemus*, and *Mizuhopecten yessoensis* GnRH-R II sequences, respectively. Phylogenetic analysis indicated that HdhGnRH-R gene was clustered with GnRH-R II of *O. vulgaris* and *O. bimaculoides*. qPCR assay demonstrated that the mRNA expression level of this receptor was significantly higher in the pleuropedal ganglion than that in any other examined tissue. Transcriptional activities of this gene in gonadal tissues were significantly higher in the ripening stage. The mRNA expression of this gene was significantly higher in pleuropedal ganglion, testis, and ovary at higher effective accumulative temperature (1000 °C). In situ hybridization revealed that HdhGnRH-R mRNA was expressed in neurosecretory cells of pleuropedal ganglion. Our results suggest that HdhGnRH-R gene synthesized in the neural ganglia might be involved in the control of gonadal maturation and gametogenesis of *H. discus hannai*. This is the first report of GnRH-R in *H. discus hannai* and the results may contribute to further studies of GPCRs evolution or may useful for the development of aquaculture method of this abalone species.

## 1. Introduction

Gonadotropin-releasing hormone receptors (GnRH-Rs) play crucial roles in mediating the effect of GnRH on the synthesis and secretion of gonadotropin hormone which acts on gonad to stimulate gametogenesis, gonadal cell proliferation, and the production of gonadal steroids [1]. Endocrine functions and neural transmissions in vertebrates are regulated by multiple GnRH-Rs, characterized by the presence of seven transmembrane domains connected by hydrophobic extracellular and intracellular loops [2]. GnRH-Rs are localized in anterior pituitary gonadotrophs/or peripheral tissues of mammalian and non-mammalian vertebrates [3]. These receptors can mediate their actions by association with G-proteins that can activate a phosphatidylinositol-calcium second messenger system to initiate cellular signaling pathways to increase the synthesis and secretion of luteinizing hormone (LH) and follicle-stimulating hormone (FSH) [4,5]. GnRH-Rs in vertebrates have been categorized into two major types (GnRH-R I and GnRH-R II) according to their binding affinities and activation sensitivities to the individual GnRH peptide [6]. In invertebrates, the GnRH-R superfamily consists of four G protein-coupled receptor family (GPCR) members: invertebrate GnRH receptor (invGnRH-R), corazonin receptor (Crz-R), adipokinetic hormone receptor (AKH-R), and AKH/Crz-related peptide receptor (ACP-R) [6]. GnRH-R superfamily in invertebrates has evolved by gene duplication as follows: amphioxus and ancestral species of protostomes and deuterostomes possess both vertebrate and invGnRH-R, mollusks, and annelids possess both invGNRH-R and AKH-R, and arthropods possess Crz-R and AKH-R [7]. Roch et al. [8] reported that the members of GnRH-R superfamily share a common ancestral gene with the vasopressin/oxytocin receptor (VP-R) and crustacean cardioactive peptide receptor (CCAP-R) superfamily.

GnRH-R transcript was first isolated and characterized from the pituitary αT3 gonadotrophe cell line of mouse. It encodes 327 amino acids containing seven hydrophobic transmembrane domains [9]. Multiple GnRH-Rs have been characterized in a number of mammalian and non-mammalian vertebrates, suggesting that most organisms might contain two or more functional GnRH-Rs in the central nervous system (CNS) [10,11,12,13,14,15,16]. GnRH-Rs of eutherian mammals have a truncated intracellular cytoplasmic C-terminal tail, indicating that the C-terminal tail might have disappeared in the evolutionary lineage between mammalian and non-mammalian vertebrates [17,18,19]. To the best of our knowledge, in mollusks, GnRH-R transcripts have been recently isolated from *O. vulgaris* [20], *Patinopecten yessoensis* [21], and *Crassostrea gigas* [22]. Oct-GnRH-R shows predominant expression in the central nervous system (CNS), peripheral nervous system, and several peripheral tissues (stomach, heart, branchia, oviduct, ovary, egg, and testis). It has been hypothesized that oct-GnRH-R can receive signals for multiple physiological and behavioral functions [20]. The py-GnRH-R like mRNA expression was found in the neural ganglia, gonad, mantle, and gill tissues. All these findings indicate that protostomian invertebrate GnRH-R can act as a key mediator in both CNS and peripheral tissues for various aspects of physiological functions [21].

The Pacific abalone, *H. discus hannai*, is an economically important marine bio-resource distributed along coastal waters of East Asia. It is deemed as one of the most valuable and popular seafood owing to the presence of bioactive molecules [23].

Several studies related to GnRH-Rs have been performed in different vertebrate and invertebrate species but to date, the characterization and expression analysis of GnRH-R in abalone species are lacking. Hence, the objective of the present study was to isolate a full-length sequence of GnRH-R and determine its expression pattern by quantitative real time PCR (qPCR) and in situ hybridization.

## 2. Results

### 2.1. Cloning and Sequence Analysis of GnRH-R Gene

**Figure 1:**
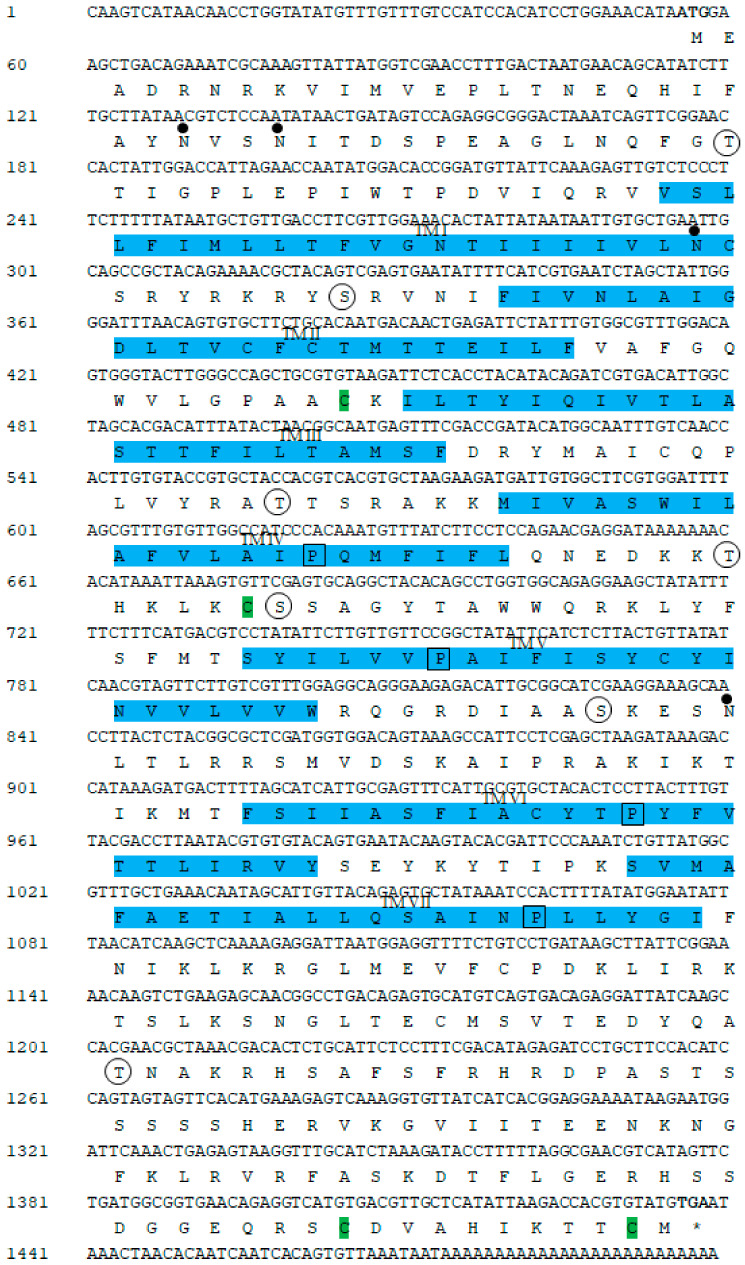
Full-length nucleotide and deduced amino acid sequences of the cloned GnRH-R gene from *H. discus hannai*. Initiation and termination codons (asterisks) are in bold font. The seven putative transmembrane domains are highlighted with light blue and indicated by roman numerals. Possible N-linked glycosylation motifs are designated by dots. Potential sites for phosphorylation are indicated by circles. Four cysteine residues (Cys-130, Cys-207, Cys-450, and Cys-459) that are likely to form intramolecular disulfide bridges are shaded in green. The proline residues that have been shown to be involved in the GnRH-R binding pocket are denoted by black boxes.

The complete cDNA sequence of GnRH-R gene was obtained from the pleuropedal ganglion, and deposited in GenBank with an accession number of MN270936. The full-length cDNA consisted of 1499-bp with a 5′-UTR (untranslated region) of 55-bp, a 3′-UTR of 61-bp, and an open reading frame (ORF) of 1383-bp (Figure 1). The ORF encoded a protein sequence of 460 amino acids, with an estimated molecular mass of 52.22 kDa and an isoelectric point (pI) of 9.57. An NCBI BLASTP analysis showed that the amino acids of GnRH-R gene in *H. discus hannai* shared 63%, 52%, and 30% sequence identities with *O. vulgaris*, *L. polyphemus*, and *M. yessoensis* GnRH-R II, respectively. In silico analysis indicated that this protein might be localized in the plasma membrane. The cloned sequence contained four potential N-linked glycosylation motifs (^25^NVS, ^28^NIT, ^81^NCS, and ^262^ NLT), and four cysteine residues (Cys-130, Cys-207, Cys-450, Cys-459) that might form two intramolecular disulfide bonds. Threonine and serine residues at positions ^42^T, ^90^S, ^168^T, ^202^T, ^208^S, ^258^S, and ^383^T serve as putative phosphorylation sites for protein kinase A or C. Hydrophobicity analysis of the deduced amino acid sequence indicated that GnRH-R gene of *H. discus hannai* possessed seven hydrophobic transmembrane domains, each of which had 20 to 23 residues. This cloned receptor also contained GnRH-R binding pocket proline residue in TM IV, V, VI, and VII.

**Figure 2:**
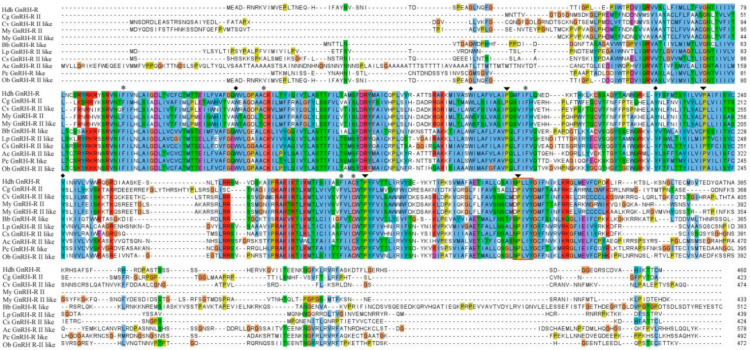
Multiple sequence alignment of HdhGnRH-R gene with representative invertebrates GnRH-R homologs. Conserved residues that might be involved in GnRH binding to the receptor are marked with asterisks. Conserved GPCRs activation residues are indicated by rectangular boxes. The conserved proline residues and tertiary structure formation residues are denoted by black arrowheads and diamond circles, respectively. Hdh-*H. discus hannai* (MN270936); Cg-*C. gigas* (EKC32751.1); Cv-*C. virginica* (XP_022304394.1); My-*M. yessoensis* (GnRH-R II: OWF54054.1; GnRH-R II like: XP_021346032.1); Bb-*Branchiostoma belcheri* (XP_019622956.1); Lp-*Limulus polyphemus* (XP_022237861.1); Cs-*Centruroides sculpturatus* (XP_023221703.1); Ac-*A. californica* (XP_005106606.1); Pc-*Pomacea canaliculata* (XP_025087144.1); Ob-*Octopus bimaculoides* (XP_014770942.1).

Alignment of GnRH-R amino acid sequence of *H. discus hannai* and other invertebrates revealed that receptor binding residues were present in conserved regions of this cloned sequence. Similar to other GPCRs, active binding amino acid residues DRY and NPXXY are also conserved in the structural profile of GnRH-R gene in *H. discus hannai* (Figure 2).

**Figure 3:**
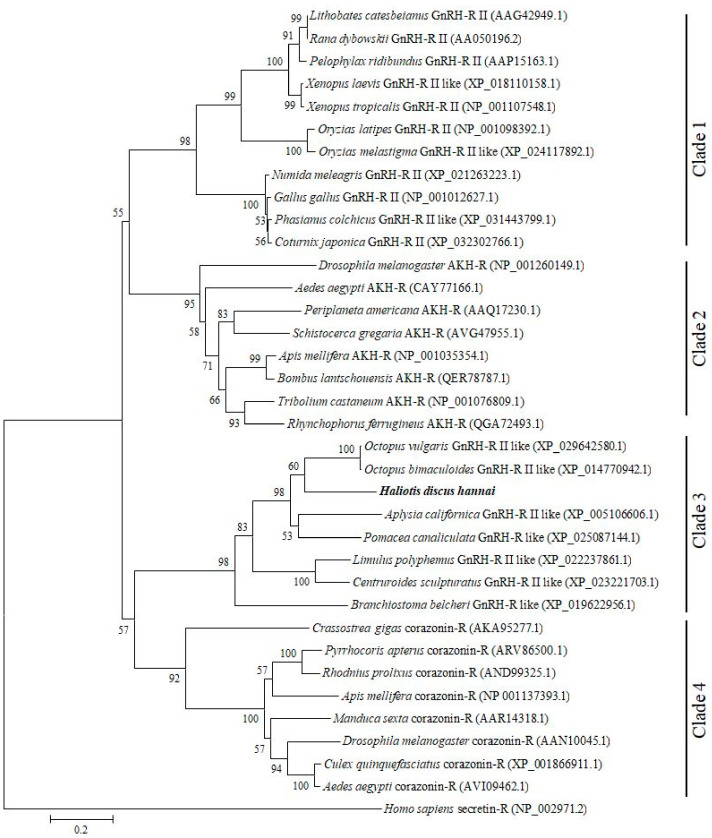
Molecular phylogenetic tree of GnRH-R and other related proteins from vertebrates and invertebrates. A phylogenetic tree was constructed from the amino acid sequences using the maximum likelihood method. Bootstrap probabilities are given at each node. The scale bar indicates an evolutionary distance of 0.2 amino acid substitutions per site. The GnRH-R gene of *H. discus hannai* is highlighted in bold font. *H. sapiens* secretin receptor was used as outgroup.

A phylogenetic tree was constructed using GnRH-Rs along with other structurally related hormone of various protostome and deuterostome species to elucidate their possible evolutionary relationships. The constructed phylogenetic tree revealed four distinct clades: GnRH-R II of vertebrates including frog, freshwater teleost, and aves clusters as clade 1; AKH-R of invertebrates including several insects assembles as clade 2; GnRH-R of several mollusk, arthropods, and amphioxus is grouped in clade 3; Crz-R of bivalve and arthropods forms clade 4. Based on analysis of the phylogenetic tree, GnRH-R gene of *H. discus hannai* is located in clade 3 and aligned with GnRH-R of *O. vulgaris* and *O.bimaculoides*, then clustered with GnRH-R of *A. californica* and *P. canaliculata* (Figure 3).

### 2.2. Tissue Expression Profile of GnRH-R mRNA

**Figure 4:**
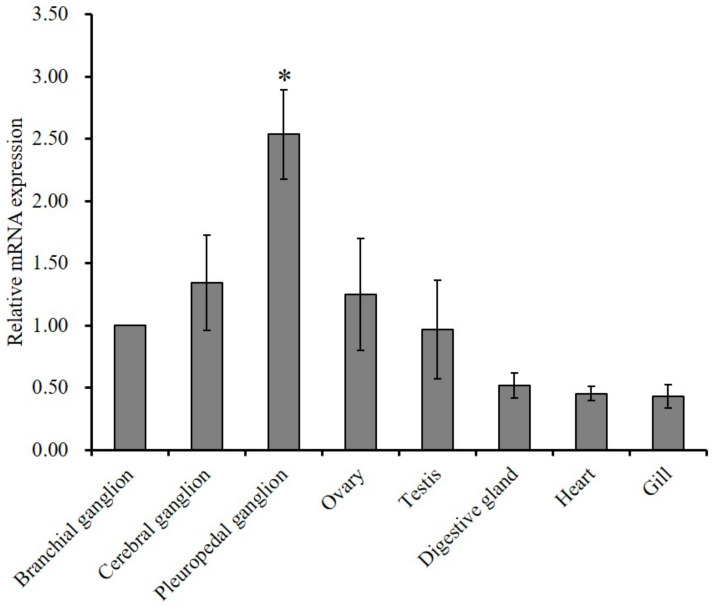
Quantitative PCR analysis of GnRH-R mRNA expression pattern (means ± SD, *N* = 3) in various tissues of abalone. Data were compared with the relative value of the branchial ganglion (1). Asterisks indicate significant differences (*p* ≤ 0.05).

Tissue specific expression and relative mRNA expression of GnRH-R mRNA were examined using quantitative polymerase chain reaction (qPCR) assay. Results of analysis revealed that the mRNA expression of GnRH-R gene was significantly (*p* ≤ 0.05) higher in the pleuropedal ganglion than in any other examined tissue (Figure 4).

**Figure 5:**
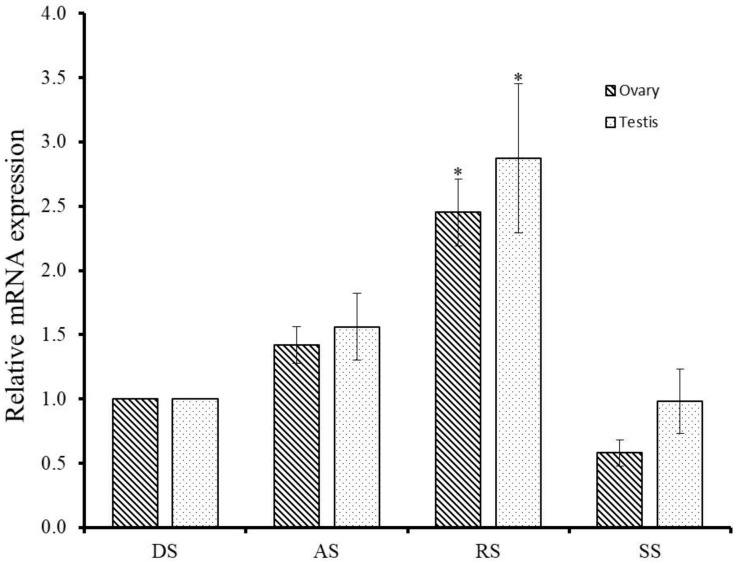
Relative mRNA expression of *H. discus hannai* GnRH-R mRNA in gonads at different stages of reproduction. The expression level in the gonad at degenerative stage is set as 1 to calibrate the expression levels in gonad at other stages. Asterisks indicate significant differences (*p* ≤ 0.05) when compared with the degenerative stage of gonad. DS-Degenerative stage; AS-Active stage; RS-Ripening stage; SS-Spent stage.

To detect the involvement of GnRH-R gene of *H. discus hannai* in the control of reproductive process, qPCR assay was performed at different gametogenesis stages. The GnRH-R mRNA transcript exhibited higher expression in the testis than in ovary at all stages of gametogenesis. Significantly (*p* ≤ 0.05) higher expression of GnRH-R mRNA was detected at the ripening stage in both male and female gonads than in other stages (Figure 5). There were no significant differences between the degenerative stage and other stages of gonad.

**Figure 6:**
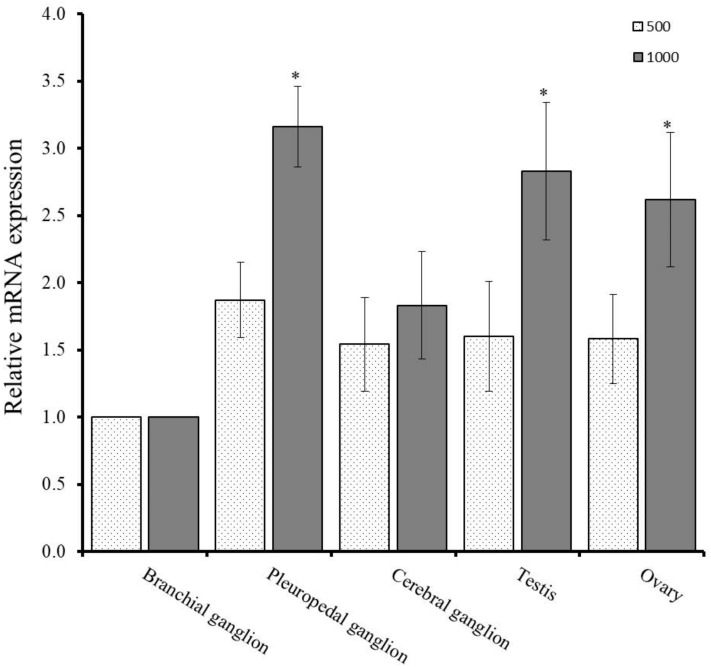
Expression levels of GnRH-R mRNA at different effective accumulative temperature (EAT) in neural ganglia and gonadal tissues as determined by qPCR assay. Data were compared with the value of the branchial ganglion, which was assigned a relative value of 1. Asterisks indicate significant differences (*p* ≤ 0.05).

Temperature is the major external environmental factor which regulate the reproductive cycle of the gastropod mollusk. Regulation of gonadal maturation and spawning of abalone is greatly influenced by effective accumulative temperature (EAT). Quantitative PCR assay was conducted to determine the changes of mRNA expression level of GnRH-R in neural ganglia and gonad at different EAT. qPCR analysis revealed that mRNA expression level of GnRH-R gene was elevated at 1000 °C in the neural ganglia and gonadal tissues than 500 °C. Significantly higher expression was recorded in the pleuropedal ganglion, testis, and ovary at EAT of 1000 °C (Figure 6).

**Figure 7:**
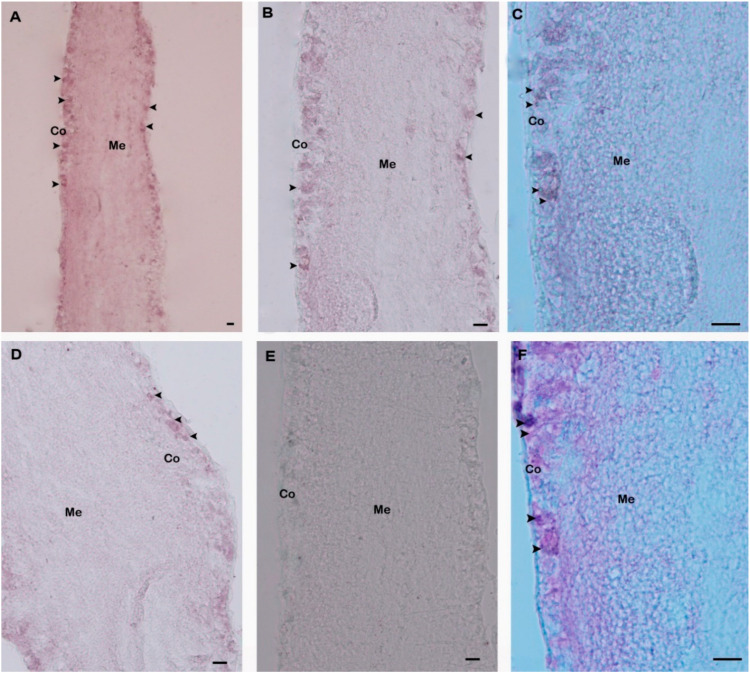
Distribution of GnRH-R mRNA in pleuropedal ganglion demonstrated by in situ hybridization. (**A**) Positive signals with an antisense probe are detected in the neurosecretory cells (arrows) of the cortex region; (**B**) Medium magnification of A; (**C**) High magnification showing hybridized GnRH-R mRNA in neurosecretory cells; (**D**) Medium magnification showing hybridization signal in the other part of cortex region; (**E**) Hybridized with the GnRH-R mRNA sense riboprobe showing no hybridization signal; (**F**) Fast red counterstaining showed hybridized neurosecretory cells. Co-Cortex; Me-Medullae. Scale bars, 100 µm.

Spatial expression and distributions of *H. discus hannai* GnRH-R gene in pleuropedal ganglion was detected using in situ hybridization. GnRH-R mRNA expressing neurons were found in the cortex of pleuropedal ganglion (Figure 7A–D). Hybridization signals were not detected in the negative control section (Figure 7E). The positive signal was likely localized in neurosecretory cells of the cortex region (Figure 7F) as revealed by fast red counterstaining.

## 3. Discussion

GnRHs exert their physiological actions through interactions with GnRH-Rs that belong to the rhodopsin-like GPCR family. In mollusks, GnRH-Rs have been identified in *O. vulgaris* [20]. Four types of GnRH-R like transcripts (Cg-GnRH-R, Cg-GnRH-RII-L, Cg-GnRH-RII-S, and Cg-GnRH-R-TF) have been reported in bivalve mollusks, *C. gigas* [22,24]. To date, the cloning and characterization of GnRH-R in abalone species have not been reported yet. For the first time, this study demonstrated the molecular architecture and expression profile of GnRH-R gene in the Pacific abalone, *H. discus hannai*. The cloned GnRH-R gene displayed several key features of a typical GPCR such as seven hydrophobic transmembrane domain containing distinctive sequence patterns, an N-linked glycosylation consensus motif, and potential phosphorylation sites of serine and threonine in the intracellular loop and C-terminal region (Figure 1) [22,25]. Similar to other GnRH-Rs, transmembrane domains of HdhGnRH-R gene are connected by three intracellular and three extracellular loops [26]. The presence of four cysteine residues that are likely to form disulfide bridges suggest that they are crucial for the packing and stabilizing of a restricted number of conformations of these seven transmembrane helical segments [27]. The phosphorylation site of the cloned receptor plays a key role for several signal transduction cascades [28]. The identified Cys-459 residues in C-terminal tail of this receptor serve as a potential site for palmitoylation. The palmitate on C-terminal cysteine residues is believed to form an additional cytosolic loop which may influence receptor mobility [29] or G protein-coupling [30].

The characteristic tripeptide DRY (Asp154-Arg155-Tyr156) at the interface of TM-III and the NPXXY (Asn335-Pro336-Tyr339) motif in TM-VII are involved in receptor activation of the GPCRs family [31,32]. Moreover, the cloned sequence possesses most of the well conserved residues found in rhodopsin-like GPCRs which mediate the tertiary structure required for their functional activity [33,34].

The outcome of multiple sequence alignment of HdhGnRH-R gene indicated that residues involved in GnRH binding to the receptors were well conserved (Figure 2). Proline residues in TM IV, V, VI, and VII of HdhGnRH-R gene might be crucial for the formation of binding pocket [35]. 

In phylogenetic analysis, GnRH-R gene of *H. discus hannai* was aligned with GnRH-R II of *O. vulgaris* and *O. bimaculoides*. Results of phylogenetic analysis suggest that our cloned gene is a potential member of the invertebrate GnRH-R superfamily. Therefore, it is designated as HdhGnRH-R gene. Rodet et al. (2005) reported that the GnRH-R of *C. gigas* (Cg-GnRH-R) is structurally similar to insect adipokinetic hormone receptor (AKH-R) and robustly clustered with fruitfly and silkworm AKH-R.

The predominant expression of GnRH-R has been reported in the brain of basal vertebrate, sea lamprey during adult stage [36]. González-Martínez et al. [16] has also detected an exclusive expression of GnRH-R in the proximal pars distalis of the pituitary gland of European sea bass. GnRH-Rs are widely expressed in the central nervous system and several peripheral tissues of *O. vulgaris* [20]. Cg-GnRH-R expression has been observed in adult gonads of both sexes and it is expected to play a role in reproduction [22]. *P. yessoensis* GnRH-R like mRNA is widely expressed in various tissues including neural ganglia [21]. In the present study, a significantly higher expression of HdhGnRH-R gene was detected in the pleuropedal ganglion than in other ganglia and gonadal tissues (Figure 4). These findings in the abalone suggest that HdhGnRH-R gene can be produced in pleuropedal ganglion and then GnRH peptide secreted from individual ganglion to their own target tissues.

HdhGnRH-R transcript showed a differential expression pattern during the gametogenesis cycle in both testis and ovary. The mRNA distributions of this transcript suggest that the reception of GnRH signal on the gonadal cells might be required during gonadal maturation. The significantly increased level of expression was found during the ripening stage in both male and female gonad (Figure 5). Results indicate that HdhGnRH-R is involved in gametogenesis and its role in testis and ovary may not be the same. A similar pattern of expression was also observed for the Pacific oyster GnRH-R [22].

Gonadal maturation of abalone is greatly influenced by effective accumulative temperature (EAT) [37]. To determine the effect of EAT on gonadal maturation, the expression pattern of HdhGnRH-R mRNA was analyzed in neural ganglia and gonad at different EAT. In the present study, significantly higher expression was found in pleuropedal ganglion, testis, and ovary at higher EAT (Figure 6) suggesting that EAT act as a triggering stimulus to synthesize GnRH-R mRNA in pleuropedal ganglion which in turn act on gonad for gonadal maturation. The results also indicated that the maturation rate and number of gametes increases with increasing EAT.

The spatial expression of GnRH-R has been detected in the brain and pituitary of sea lamprey and European sea bass using in situ hybridization [16,36]. An exclusive mRNA expression of Oct-GnRH-R gene has been detected in the palliovisceral lobe and superior buccal lobe of brain in *O. vulgaris* [20]. Our report offers the first description of the expression pattern of GnRH-R transcript in abalone using ISH. In the present study, the existence of a GnRH-R transcript was detected in neurosecretory cells of pleuropedal ganglion.

## 4. Materials and Methods

### 4.1. Experimental Animals and Tissue Collection

Two-year-old adult female abalone (shell length of 10.5 cm and body weight of 148.2 g) used in the present study was collected from Naesan, Gogun-myeon, Jindo Island (34°31′16.2″ N 126°22′28.7″ E) and brought to the laboratory in the Department of Fisheries Science, Chonnam National University. Neural ganglia (pleuropedal, cerebral, and branchial ganglion), ovary, digestive gland, heart, and gill were retrieved from the abalone after anesthetization. Tissue samples were frozen immediately in liquid nitrogen and stored at −80 °C for total RNA isolation.

To prepare cryosections, the pleuropedal ganglion was washed in phosphate buffered saline (PBS; pH 7.4), and immersion overnight in 4% (*w/v*) paraformaldehyde (PFA). Next, tissues were washed with PBS for 1 min each time to drain off excess 4% PFA. Tissues were then transferred into 30% sucrose and kept overnight at 4 °C. Afterwards, tissues were embedded using a frozen section compound (FSC 222, Leica Biosystems, Wetzlar, Germany) and stored at −20 °C for subsequent use. Sectioning (8 µm in thickness) was done using a cryostat device (CM 3050; LEICA, Wetzlar, Germany) and placed on electrostatically charged slides (SuperFrost Plus; VWR International, Radnor, PA, USA). Slides were allowed to air-dry for 30 min and then stored at −20 °C until use.

All animal experimentation was performed in accordance with the guidelines of the Institutional Animal Care and Use Committee of Chonnam National University (CNU IACUC) and according to Article 14th of the Korean Animal Protection Law of the Korean government. No specific permissions are required to work with abalone in South Korea. Similarly, no permissions were needed for the collection of *H. discus hannai* from sample sites because they were not collected from the protected area and this species is not an endangered or protected species.

### 4.2. RNA Isolation and cDNA Synthesis

RNAs were isolated from various tissues of Pacific abalone using an RNeasy mini kit (Qiagen, Hilden, Germany) based on the manufacturer’s protocol. Genomic DNA was eliminated by treatment with RNase-free DNase (Promega, Madison, WI, USA). Concentration, and purity of RNA were determined with a spectrophotometer (NanoDrop^®^ NP 1000 spectrophotometer) and gel electrophoresis. Total RNA (1 µg) served as templates to synthesize first strand cDNA using a Superscript^®^ III First-Strand synthesis kit (Invitrogen, Carlsbad, CA, USA) according to the manufacturer’s protocol.

### 4.3. cDNA Cloning of GnRH-R Gene

To perform molecular cloning of GnRH-R gene, primers (sense: 5′-CAAACTCATGGTTACACTGG-3′ and antisense: 5′-GCAGAAGACCTCCATGAGT-3′) were designed based on predicted nucleotide sequences of *O. bimaculoides* (GenBank accession no. XM_014915460.1). Phusion^®^ High-Fidelity DNA Polymerase (Biolabs Inc., New England) was used in reverse transcription-polymerase chain reaction (RT-PCR). PCR was performed in a 20 μL reaction volume containing 1 μL (20 pmol) each of forward and reverse primers, 4 μL of 5× Phusion HF buffer (1×), 2 μL of dNTP (200 μM), 0.5 μL of 1 U Phusion DNA polymerase, 10.5 μL sterile distilled water (dH_2_O), and 1 μL cDNA from pleuropedal ganglion as template. PCR cycling program consisted of a denaturation at 94 °C for 3 min, followed by 35 cycles at 94 °C for 2 min, 58 °C for 30 s, 72 °C for 30 s, and a final dissociation step at 72 °C for 5 min. Resultant amplicons were purified using a Wizard SV gel and PCR Clean-Up kit (Promega). Purified PCR products were then ligated into pTOP Blunt V2 vector (Enzynomics, Daejeon, Korea) and transformed into competent *Escherichia coli* DH5α cells (Enzynomics). Positive clones were purified using a plasmid mini kit (Qiagen) and sequenced by Macrogen Online Sequencing System (Macrogen, Seoul, Korea).

The full-length cDNA sequence was found using a SMARTer^®^ RACE 5′/3′ Kit (Clontech Laboratories, Inc.,Mountain View, CA , USA) according to the manufacturer’s recommendation. 5′- and 3′-RACE cDNA was synthesized using 1 μg of total RNA from the pleuropedal ganglion of Pacific abalone according to the kit manual. Gene-specific primer sequences (GSPs), including a 15-bp overlap with the 5′-end of the GSP sequence (antisense primer: 5′- GATTACGCCAAGCTT GTAGGTGAGAATCTTACACGCAGCTGGC-3′, sense primer: 5′- GATTACGCCAAGCTTTACACAGCCTGGTGGCAGAGGAAGC-3′), were used to conduct 5′- and 3′-RACE PCR, respectively. RACE PCR was performed in a 50 μL reaction volume containing 15.5 μL PCR grade water, 25 μL 2× SeqAmp buffer, 1 μL SeqAmp DNA Polymerase, 5 μL 10× universal primer mix (UPM), 1 μL of 5′ or 3′ GSP primer (10 µM), and 2.5 μL cDNA as template. Touchdown PCR was employed for 25 cycles for 3′-RACE and 35 cycles for 5′-RACE PCR following the manufacturer’s instructions. RACE PCR products were purified using NucleoSpin Gel and PCR Clean-Up kit. Afterwards, purified products were ligated into a linearized pRACE vector, transformed into stellar competent cells, and then sequenced as described previously. Finally, these sequenced RACE products were assembled by overlapping with the initial fragment.

### 4.4. Bioinformatics Analysis

The GnRH-R protein sequence of *H. discus hannai* was analyzed with the BLAST algorithm at NCBI (https://blast.ncbi.nlm.nih.gov/Blast.cgi). The protein transmembrane domain was determined with TMHMM server 2.0 (http://www.cbs.dtu.dk/services/TMHMM/). Phosphorylation sites (serine/threonine) and N-linked glycosylation sites of the protein were predicted using NetPhosK 3.1 (http://www.cbs.dtu.dk/services/NetPhos/) and NetNGlyc1.0 server (http://www.cbs.dtu.dk/services/NetNGlyc/), respectively. Its physical and chemical properties were determined using ProtParam (http://expasy.org/tools/protparam.html). Protcomp (http://www.softberry.com/berry.phtml) was used to determine subcellular localization of the protein. Multiple alignments of the GnRH-R protein sequences of *H. discus hannai* and other invertebrate species were performed using Clustal Omega [38,39]. Jalview, version 2.10.0 (www.jalview.org) was used for editing and visualizing protein sequence alignment [40]. For phylogenetic analysis, invertebrate and vertebrate sequences of GnRH-R, adipokinetic hormone receptor (AKH-R), and corazonin receptor (Crz-R) were retrieved from NCBI database using the BLASTP program. The tree was constructed with MEGA software (version 6.0, Research Center for Genomics and Bioinformatics, Tokyo, Japan) using a maximum likelihood based approach with 1000 bootstrap trials [41].

### 4.5. Quantitative PCR Expression Analysis

GnRH-R mRNA expression was analyzed by Quantitative real-time PCR (qPCR) assay using cDNA from a variety of tissues of *H. discus hannai* of both sexes (n = 12, shell length: 9.12 ± 0.8 cm, body weight: 83.68 ± 3.97). qPCR assay was carried out using a 2× qPCRBIO SyGreen Mix Lo-Rox kit (PCR Biosystems Ltd., London, UK) to determine the expression level of the GnRH-R gene in different tissues. A pair of primers (forward: 5′-CAACTGAGATTCTATTTGTGGC-3′ and reverse: 5′-AGCCACAATCATCTTCTTAGC-3′) designed from *H. discus hannai* GnRH-R cloned sequence were used to conduct qPCR. Ribosomal protein L5 (RPL-5) (GenBank accession no. JX002679.1) was used as a reference gene for this experiment [42]. Its primers were: forward, 5′-TGTCCGTTTCACCAACAAGG-3′; reverse, 5′-AGATGGAATCAAGTTTCAATT-3′. The PCR reaction volume was 20 μL, containing 1 μL cDNA template, 1 μL (10 pmol) of each forward and reverse primer, 10 μL SyGreen Mix, and PCR grade water to make up the volume. Amplification conditions were as follows: pre-incubation at 94 °C for 3 min, followed by 40 cycles of three-step amplification at 94 °C for 2 min, 60 °C for 30 s, and 72 °C for 1 min. Three replications were used for each sample. Relative mRNA levels of GnRH-R gene were analyzed using the cycle threshold 2^−ΔΔct^ method [43].

### 4.6. Gonadal Expression of GnRH-R mRNA during Gametogenesis

Expression levels of GnRH-R mRNA in gonads at different reproductive stages were determined using the qPCR method. Reproductive stages of gonads were categorized as our previous report [44]. One microliter cDNA template of different stages was used for qPCR as described previously.

### 4.7. Effects of Effective Accumulated Temperature (EAT) on GnRH-R mRNA in Neural Ganglia and Gonad

Collected abalones were exposed to 9.5 °C of filtered sea water for one month with continuous aeration. The sample preparation and qPCR assay was conducted according to the method described by Sharker et al. [45]. The abalones were reared for 109 days and 157 days for obtaining the effective accumulated temperature (EAT) at 500 °C and 1000 °C, respectively. One microliter of cDNA template each from neural ganglia (pleuropedal, cerebral, and branchial) and gonadal tissues (ovary and testis) were used for qPCR assay.

### 4.8. In Situ Hybridization (ISH)

For in situ hybridization, primer pairs (forward: 5′-TAACAGTGTGCTTCTGCAC-3′; reverse: 5′-CGTAGAGTAAGGTTGCTTTC-3′) were designed for a 487-bp amplification fragment obtained from the pleuropedal ganglion. The amplicon was ligated with a pGEM-T Easy vector (Promega, Madison, WI, USA) and the plasmid was sequenced to confirm the identity and orientation of the product. DIG-labeled oligonucleotides for antisense and sense RNA probes were synthesized by in vitro transcription with T7 RNA polymerase (New England BioLabs, Ipswich, MA, UK) and SP6 RNA polymerase (Promega) using a digoxigenin (DIG) RNA labeling mix (Roche, 68,298 Mannheim, Germany) according to the manufacturer’s protocol.

ISH was carried out according to a method described previously [44,45,46]. Briefly, tissue sections were pre-hybridized with hybridization buffer and yeast total RNA (50 μL) for 2 h and then hybridized with the RNA probe at 65 °C overnight. After hybridization, tissue sections were incubated with alkaline phosphatase-conjugated anti-digoxigenin-Ap, Fab fragments antibody (diluted 1:500 in blocking solution [Roche]) at 4 °C overnight. Subsequently, the sections were washed with PBST, rinsed in alkaline tris buffer and finally treated with the labeling mix (2 mL alkaline tris buffer, 9 μL nitroblue tetrazolium, 7 μL 5-bromo-4-chloro-3-indoly phosphate disodium salt) to observe the color. Sections were viewed using a stereo microscope (SMZ1500, Nikon, Tokyo, Japan).

### 4.9. Nuclear Fast Red Counterstain

Counterstaining was performed with nuclear fast red using antisense probe hybridized slides. These slides were then washed several times using distilled water and treated with nuclear fast red solution (Sigma-Aldrich, St. Louis, MO, USA) for 5 min. Dehydration was accomplished by using a graded series of ethyl alcohol (70–100%) for 2 min. Finally, slides were dipped in histo-clear (National diagnostics, Atlanta, USA) for 3 min, cover-slipped using Permount mounting medium, and viewed under a stereo microscope.

### 4.10. Statistical Analysis

Statistical analyses of GnRH-R mRNA expression in different tissues and gametogenetic stages were performed using SPSS software version 16.0, followed by Tukey’s multiple comparisons test to determine statistical differences. A *p*-value of ≤0.05 was considered as statistically significant.

## 5. Conclusions

A full-length cDNA encoding HdhGnRH-R gene was cloned from the pleuropedal ganglion of the Pacific abalone and its spatiotemporal mRNA expression level was analyzed. Results of in situ hybridization indicated that HdhGnRH-R gene is present in neurosecretory cells of pleuropedal ganglion which could be the principal site of their production. Moreover, a differential expression of HdhGnRH-R transcript in the gonadal tissues during reproductive cycle was found, suggesting that this receptor could contribute to the control of reproductive processes. These preliminary investigations will contribute to a better understanding of neuroendocrinological regulation in abalone. However, further works are necessary to clarify the physiological regulatory mechanisms of the GnRH-R in abalone.
